# Circulating Cell-Free DNA Combined to Magnetic Resonance Imaging for Early Detection of HCC in Patients with Liver Cirrhosis

**DOI:** 10.3390/cancers13030521

**Published:** 2021-01-29

**Authors:** Marianna Alunni-Fabbroni, Sabine Weber, Osman Öcal, Max Seidensticker, Julia Mayerle, Peter Malfertheiner, Jens Ricke

**Affiliations:** 1 Department of Radiology, University Hospital, LMU Munich, 81377 Munich, Germany; Osman.Oecal@med.uni-muenchen.de (O.Ö.); Max.Seidensticker@med.uni-muenchen.de (M.S.); Peter.Malfertheiner@med.uni-muenchen.de (P.M.); Jens.Ricke@med.uni-muenchen.de (J.R.); 2 Department of Medicine II, University Hospital, LMU Munich, 81377 Munich, Germany; Sabine.Weber@med.uni-muenchen.de (S.W.); Julia.Mayerle@med.uni-muenchen.de (J.M.)

**Keywords:** liver cirrhosis, MRI, HCC, liquid biopsy, circulating cell-free DNA

## Abstract

**Simple Summary:**

Liver cirrhosis can develop into malignant disease over time. Frequent monitoring would be advisable to detect the earliest signs of HCC progress resulting in a possible earlier treatment of the patient. Our study showed that the combination of genetic analysis of DNA freely circulating in the blood of the cirrhotic patients with MRI can represent a powerful strategy to timely identify suspect lesions, which can then be followed up more closely and thus potentially be treated earlier. In this way, personalized medicine can be applied to liver diseases such as cirrhosis.

**Abstract:**

Liquid biopsy based on circulating cell-free DNA (cfDNA) is a promising non-invasive tool for the prognosis of hepatocellular cancer (HCC). In this exploratory study we investigated whether cfDNA and gene variants associated with HCC may be found in patients with liver cirrhosis (LC) and thus identify those at an increased risk for HCC. A cohort of 40 LC patients with no suspect neoplastic lesions was included in this study. Next generation sequencing (NGS) of cfDNA isolated from plasma was performed on a panel of 597 selected genes. Images of the patients who underwent MRI with hepatospecific contrast media during the study period were retrospectively re-evaluated (imaging was not part of the prospective study). cfDNA was detected in the plasma of 36 patients with LC. NGS-based analyses identified 20 variants in different combinations. Re-evaluation of the MRI images that were available for a proportion of the patients (*n* = 27) confirmed the absence of lesions in 8 cases carrying cfDNA without variants. In 6 of 19 patients with identified variants and MRI images available, MRI revealed a precursor lesion compatible with HCC and new lesions were discovered at follow-up in two patients. These precursor lesions were amenable for curative treatments. Mutation analysis revealed selective HCC related gene mutations in a subset of patients with LC, raising the suspect that these patients were at an increased risk for HCC development. MRI findings confirmed suspect nodular lesions of early stage HCC not detected with current standard screening procedures, which were only seen in patients carrying cfDNA variants. This opens a perspective for an HCC screening strategy combining both liquid biopsy and MRI in patients with LC.

## 1. Introduction

Hepatocellular carcinoma (HCC) is the third leading cause of cancer-related death globally [1,2,3,4] with a 5-year survival rate lower than 10% [5]. The high mortality rate is mainly due to the underlying liver disease and especially at a late diagnosis, when curative treatment can no longer be offered. If patients are detected with early stage HCC, several curative treatment options are available, including surgical resection, transplantation and microinterventional therapy, which can increase the 5-year survival rate up to 70% [6]. Liver cirrhosis (LC) is the underlying preneoplastic condition in 90% of patients with HCC in the western world [7]. The cirrhotic liver takes several steps in its progression to HCC by evolving from a chronic inflammatory-fibrotic stage, through low-grade and high-grade dysplastic nodules, eventually leading to neoplasia. The sequence of events offers the opportunity to capture the neoplastic development at an early stage and has been translated into current clinical recommendation of screening and surveillance for HCC. In patients with LC, standard screening/surveillance based on ultrasound (US) and alpha-fetoprotein (AFP) is suboptimal, because it is lacking in sensitivity and specificity [4,8]. Magnetic resonance imaging (MRI) represents the gold standard in the diagnosis of HCC, however, due to the operating costs, its use in clinical practice is mostly reserved for patients with suspect lesions. Furthermore, MRI has its limitation in detecting malignant lesions with a diameter less than 10 mm [7,9] with accepted imaging criteria for the diagnosis of HCC (wash in and wash out) being characterized by a high specificity (close to 100%) but low sensitivity (around 70%). Therefore, mainly precursors or early forms of HCC are missed through these criteria. Supporting evidence for detection and characterization of HCC in its earlier stages through multiparametric MRI is growing, therefore the need for novel biomarkers to identify patients with LC who are at higher risk for developing HCC is urgent. Circulating cell-free DNA (cfDNA) is released in blood as a consequence of cellular apoptosis or necrosis. Although detectable at a low concentration in healthy individuals, cfDNA is found in significantly higher concentrations in patients with chronic inflammatory or malignant diseases [10,11]. cfDNA has gained considerable attention as a novel tumor biomarker: kinetic analysis [12,13] and molecular profiling [14] have demonstrated both a predictive and prognostic value in different types of malignancies [15,16,17,18]. Detectable cfDNA in a considerable amount has been reported in HCC patients, and in patients with LC [19,20,21,22]. The potential for the analysis of cfDNA variants carried by cirrhotic patients may indicate abnormal changes towards a malignant development at a stage when HCC is not yet recognizable by standard imaging. We recently performed a mutation profiling of cfDNA isolated from advanced HCC patients and identified a panel of 19 genes carrying defined mutations suggesting a possible role as driver genes and prognostic markers [23]. In this exploratory prospective cohort study, we performed mutation profiling of cfDNA isolated from LC patients without HCC according to US and AFP. The molecular profiles were compared with the findings in the multiparametric MRI imaging using hepatocyte specific contrast media. In addition, we present selected case reports to demonstrate how liquid biopsy can support imaging analysis. The aim of this study was to unravel a prognostic significance of cfDNA with respect to the LC progress.

## 2. Results

### 2.1. Clinicopathological Characteristics of the Patients

This exploratory study included 40 LC patients, with no diagnosis of HCC at the time of inclusion. Clinicopathological characteristics were collected prospectively while patients were hospitalized. Blood samples were withdrawn at the time of inclusion in the study. The clinical characteristics of the patients are summarized in Table 1.

### 2.2. Quantification of cfDNA in Cirrhotic Patients and Healthy Donors

cfDNA was isolated from plasma obtained from the LC patients. Four of the 40 cfDNA samples were excluded from the subsequent next generation sequencing (NGS) analysis due to high genomic DNA contamination or because the concentration was too low. In the remaining 36 samples, the amount of cfDNA ranged between 9.35 and 243.10 ng/mL with a mean value of 42.27 (±42.42) ng/mL (data not shown). Following the same procedure, cfDNA was also extracted from the plasma collected from HD with no known history of liver disease. Three of the 10 cfDNA samples from HD were excluded due to high genomic DNA contamination or because the concentration was too low. In the remaining seven HD samples, the amount of cfDNA ranged between 8.43 and 11.72 ng/mL with a mean value of 8.46 (±1.47) ng/mL.

### 2.3. Somatic Mutation Analysis

Genomic profiling of cfDNA was undertaken with the screening of 597 cancer-relevant genes. Only those mutations proven to have clinical impact according to the ClinVar database (National Center for Biotechnology Information—NCBI) were further analyzed. In total 20 unique variants, including single nucleotide variants (SNVs) or insertions and deletions (InDels) were discovered in our dataset, while no gene fusion was identified (shown in Table 2).

Among the 36 LC patients, 27 (75%) showed at least one variant (median = 2 variants per patient, range 1–4), with patterns changing within the patient’s group, while in 9 patients (25%) cfDNA was not showing any variant (shown in Table 3).

Among the 20 gene mutations, 3 were displaying the highest mutation allele frequency (MAF): HNF1A (*n* = 10, 28%), BAX (*n* = 9, 25%) and ASXL1 (*n* = 7, 19%). To a lower extent, variants were detected in CDH2 (*n* = 4, 11%), CYP2B6 (*n* = 2, 5%), FLCN (*n* = 2, 5%) and NBN (*n* = 2, 5%), while all the others mutations were found in single cases (*n* = 1, 3%).

### 2.4. Imaging Features in Patients Carrying cfDNA Variants

**Figure 1:**
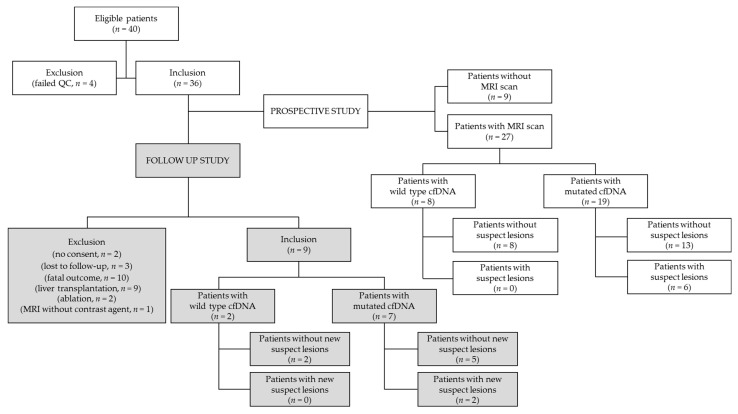
Flow chart showing the distribution of LC patients with respect to patients’ inclusion/exclusion, cfDNA analysis and imaging results in the primary prospective (white rectangles) and in the follow-up (grey rectangles) studies.

Of the 36 patients for whom cfDNA profiling was performed, 6 had no MRI scans available, and 3 underwent MRI without contrast medium. The remaining 27 patients were eligible for a scan re-evaluation performed by two independent, blinded radiologists (Figure 1).

In the patients with wild type cfDNA (*n* = 8), the presence of premalignant or malignant lesions were excluded by the initial and also the second radiological evaluation. In the remaining 19 patients, for whom NGS put in evidence different variants, the re-evaluation of the available images did not identify any suspect lesion in 13 patients, confirming the previous diagnosis. However, in six cases the new assessment identified lesions, which were then classified as high-grade dysplastic nodules (HGDN), early HCC or HCC (according to the imaging criteria described in the Image Analysis section). Thus, cfDNA variants could accurately predict HCC lesions in 6 of 19 patients, while HCC lesions could be excluded in all of the 8 patients without variants, leading to a specificity of 38.1% but sensitivity as high as 100%. A summary of the combined assessment based on imaging evaluation and cfDNA analysis is given in Table 4.

Regarding the patients with HCC, precursor lesions were already present in the scans obtained months or years before. Yet, based on size and hemodynamic features, such as arterial enhancement and venous wash-out, those lesions were not classified as overt HCC, and thus no biopsy was performed. The patients were then advised for a routine imaging follow-up. It is noteworthy that in all the cases cfDNA had already displayed specific mutations several months before the second evaluation and diagnosis. A detailed description of the single clinical cases is given in the supplementary data (Supplementary File 1 and Figures S2–S7).

### 2.5. Follow-Up Study

In order to evaluate the prognostic value of the identified variants, a follow-up study was started after 17 ± 2 months from the first blood collection and MRI re-evaluation. However, of the original cohort (*n* = 36), 27 patients could not be included (10 patients received either liver transplantation or tumor ablation, 9 died, 3 refused to participate, 4 patients were lost due follow-up and for 1 patient MRI was run without contrast media due to renal failure and therefore the identification of HCC was not possible, as summarized in Figure 1). For the remaining nine patients, a new MRI was obtained between July and September 2020: an absence of lesions was confirmed in four of those patients carrying variants and in two patients carrying wild type cfDNA (Table 5). In the patient carrying HGDN, no additional malignant lesions were observed. However, new suspect lesions were discovered in two patients whose variants had been detected. In patient 92502 with previously diagnosed HCC, a new CT and MRI revealed five new HCC lesions in both liver lobes. Retrospective evaluation of a previous MRI revealed that these lesions were already present, but that they were all hyperintense in native T1 sequences and did not have arterial hypervascularity or venous wash-out, and were therefore classified as dysplastic nodules (Figure S8 and Table 5).

In patient 92381 diagnosed with early HCC, the follow-up MRI confirmed a minimal size increase with the same imaging characteristics as before. However another 17 mm lesion in segment 7 was noticed. A retrospective evaluation of the previous MRI showed the lesion had only been 7 mm and hyperintense in all T1 weighted series without any enhancement in subtracted images, resulting in its classification as a non-malignant dysplastic nodule. Subtraction images in the follow-up MRI revealed development of arterial hypervascularity (wash-in). CT confirmed these findings and showed venous wash-out of the lesion. The newly discovered lesion was classified as HCC (Figure S2 and Table 5). Accordingly, in the follow-up study, a variant detected new suspect lesions in 2 out of 7 patients, while malignant lesions were excluded in both patients with wild type cfDNA.

### 2.6. Association of cfDNA Levels and Variants with Patient’s Clinicopathological Characteristics

cfDNA was extracted from three groups of donors: those with LC with no presence of malignant or premalignant lesions (*n* = 30), those with LC and detection of malignant hepatic lesion (*n* = 6) and healthy donors (HD) with no history of liver disease (*n* = 7). The cfDNA concentration in LC patients was found to be significantly higher than in HD (*p* < 0.005). The same result was observed if the cfDNA values that were measured in the patients diagnosed positive for HGDN, early HCC or HCC (*n* = 6) were excluded from the analysis (*n* = 30, *p* < 0.005). In a similar way the cfDNA level in the six patients carrying the malignant lesions (*n* = 6) were significantly higher than those measured in HD (*p* = 0.005). On the contrary, no significant difference (*p* = 0.467) was found when the level of cfDNA from the six patients carrying the malignant lesions was compared to the level of cfDNA in cirrhotic patients (shown in Table 6).

Due to the single time point blood collection, it is not possible at the moment to unravel an increase in the cfDNA concentration in those patients carrying the lesions with respect to the LC patients. However, in the follow-up study this point will be addressed.

Furthermore, no significant correlation was found between the amount of cfDNA and the clinical characteristics of the patients, with the exception of AFP (*p* = 0.024) (Table 7).

When the cohort was subdivided in patients with only LC (*n* = 30) and patients with HGDN, early HCC or HCC (*n* = 6), no significant association was found between the amount of cfDNA and the clinical characteristics of the patients (all *p* value ≥ 0.05). We also did not find significant differences in the molecular profiles based on their clinical characteristics. However, we observed a trend between the presence of the variant in ASXL1 and alcohol as the etiological factor (*p* = 0.088), and the presence of variants in HNF1A and gender (*p* = 0.096) or BMI (*p* = 0.064). The distribution of the variants with respect to etiologies showed that patients with alcoholic cirrhosis (AC) were those carrying the highest number of variants (9/20, 45%), while patients positive for HBV/HCV were carrying only one single mutation (1/20, 5%). Furthermore, patients with Child–Pugh A (12/20, 60%) and B (13/20, 65%) showed at least 2.5 times more variety in the variants with respect to Child–Pugh C patients (5/20, 25%) (Table 8). A solid conclusion of the significance of the correlation between variants’ detection and etiologies could not be reached due to the small number of patients included in the cohort. However, these findings should be considered as hypothesis generating and should be confirmed in larger validation studies.

## 3. Discussion

In the vast majority of cases and up to 90% in western countries, HCC develops from a cirrhotic liver [24,25]. Long-term follow-up studies report that 1.3% of patients with LC progress to HCC per year [26], supporting the importance of tight surveillance for this group of patients [27]. In addition, early detection of HCC reduces mortality by approximately 37%, mainly due to increased eligibility for resection and other therapeutic modalities with curative potential [28]. It is therefore of high priority to identify reliable tumor biomarkers for early detection of HCC. Current guidelines suggest surveillance with abdominal US with or without AFP every six months [29,30]. Although US has acceptable sensitivity (58–89%) and specificity (90%) as a surveillance test [31], it fails to detect nearly 40% of early-stage HCC cases [32]. MRI is the current imaging gold standard and a suspect nodular lesion in US is confirmed as malignant when criteria including lesion size (≥10 mm) and specific hemodynamic features (such as arterial enhancement and venous wash-out) are met. However, although the sensitivity of MRI for ≥20 mm lesions increased from 76% to 96% with the implantation of new criteria [33], the detection of smaller lesions such as those found in the first development stages of the tumor still poses a challenge. In addition, it is not uncommon that small HCC lesions in the cirrhotic liver do not meet the criteria for the arterial enhancement and the venous wash-out. In lesions of 1–2 cm, typical wash-in and wash-out criteria have a sensitivity of 71% [6]. Therefore, additional parameters for risk stratification and patient allocation for intensified follow-up with MRI using modern HCC criteria are needed. Liquid biopsy could support the monitoring of HCC development from LC, especially by way of easy access to blood samples, without the need of invasive samples collection. We previously reported a panel of distinct genetic variants in circulating tumor DNA isolated from the plasma of HCC patients, which could support the stratification of the patients with respect to the therapy response [23]. In this study we extended the analysis of cfDNA to patients with LC and with no sign of malignancy during the screening process. cfDNA has already been demonstrated as a suitable noninvasive biomarker for risk prediction of different pathologies including cancer and has also been associated to different liver diseases such as non-alcoholic fatty liver disease [34] or hepatitis B [35]. However, the value of genetic analysis of cfDNA in the surveillance of LC patients has not been explored in detail as of yet [19]. We found that cfDNA was detectable in LC patients, in HCC patients and in HD, however the levels in the first two groups were significantly higher than in the third group. On the contrary, although the mean concentration of cfDNA in the six patients carrying malignant lesions was higher than in LC patients, no statistical significance was reached. On a total of 36 cfDNA, a panel of 20 genetic variants was found in 75% (*n* = 27) of the samples analyzed, with the remaining 25% (*n* = 9) carrying no variants. In homology to HCC, frameshift mutations were detected at higher frequency in a limited number of genes including HNF1A (28%), BAX (25%) and ASXL1 (19%), with the codon changes identical to those found in HCC. BAX belongs to the Bcl-2 gene family of proapoptotic proteins and frameshift mutations correlate to cancer development in different type of organs [36,37]. HNF1A codes for the transcription factor HNF1α, which regulates hepatocyte functions; it is frequently mutated in benign hepatocellular adenomas [38,39] and its inactivation indicates a role as a tumor suppressor [40,41,42]. ASXL1 is a scaffold protein and variants have been found in different types of cancers, such as in breast [43], hematological cancers [44] and prostate cancer [45], conferring to this protein the role of a tumor suppressor [46]. At the time of blood analysis, patients were only diagnosed with LC without suspicion of any hepatic malignancy. However, in patients carrying the variants re-evaluation of the imaging scans revealed suspicious liver lesions in six (22%) cases, with four of those patients even showing progression to HCC. Importantly, mutations were detected before progression of precursor lesions into HCC. On the contrary, none of the patients with wildtype cfDNA (*n* = 8) showed signs for any type of malignant lesion in the MRI. NGS analysis displayed a sensitivity of 100%, due to the ability to identify variants at an allele fraction of <1% in plasma samples [47]. One year after the first MRI re-evaluation, a follow-up study with a second MRI was performed, to monitor the further development of lesions over time in the same patients group. Since the 5-year cumulative risk for the development of HCC in patients with LC ranges between 5% and 30%, depending on etiology, ethnicity and stage of LC [28], we were not expecting to find many pronounced changes in the MRI scans. Nevertheless, in two patients new HCC lesions were discovered in the ground of dysplastic nodules. As evidenced from the retrospective MRI evaluation, the nodules were already present but not classified as malignant when cfDNA analysis was performed. These new findings further support the prognostic role of cfDNA in the identification of early lesions in LC. This holds true especially for HGDN (which may contain HCC islets, which are difficult to be detected by tissue biopsy [25]) and for lesions smaller than 10 mm with a typical HCC enhancement pattern and for lesions without arterial hyper vascularity but venous wash-out (described as early-HCC), for whom non-invasive diagnosis and optimal treatment strategies have not yet been established. The correlation between the presence of variants and the discovery of malignant lesions based on MRI imaging highlights the importance of liquid biopsy in HCC early diagnosis. NGS analysis of cfDNA could represent a helpful supplementary test in combination with modern MRI to detect HCC and its earlier forms not only through perfusion criteria but also through other sequences, therefore providing a higher sensitivity while maintaining a high specificity as compared to HCC detection based on perfusion criteria alone. Although the heterogeneous etiology of LC and HCC poses a challenge in the definition of a driver mutation panel [48], we detected only a few (and at low frequency) of the established HCC driver mutations such as TP53, CTNNB1, KRAS, PIK3CA or TERT. These variants are usually detectable in liver cancer or in the case of preneoplastic lesions, but not in LC, as in the present study [49,50]. Concerning the three patients carrying a small HCC lesion at the time of blood analysis, it should be noted that blood sampling was performed at a very early stage of the tumor development. Since the amount of circulating tumor DNA (the quote of cfDNA derived from the tumor itself) is less than 0.1% of the total cfDNA, we could have faced a technical issue linked to a limit in the detection [51]. This study has some limitations. First, the size of the cohort is small. However, since this work is an exploratory pilot study, a validation study with a larger cohort will be necessary to confirm these preliminary results and to support their clinical significance. Secondly, we observed a relatively high rate of patients lost to follow-up, which is due also to the large number of patients dying (in this study *n* = 10), undergoing liver transplantation (*n* = 9) or receiving additional therapies (*n* = 2) as a consequence of the natural course of the disease in patients with advanced LC. For future studies, the high rate of patients lost at follow-up will be considered for the sample size estimation. In addition, the cohort was quite heterogeneous regarding the etiology of LC, which is however also reflecting the clinical reality. In the validation study we plan to base the inclusion criteria on the etiology in order to minimize the heterogeneity or to conduct a subanalysis on the basis of the different etiologies of LC. Moreover, no direct comparison between the profiles of cfDNA and corresponding biopsies was possible since tissue collection had not been performed. However as pointed out by others [22], this work reflects clinical practice, where LC and HCC monitoring are commonly based on imaging analysis without tissue diagnosis.

## 4. Materials and Methods

### 4.1. Study Population

A total of 40 patients affected by LC with no sign of cancer (median age, years ± SD: 56 ± 10.41, range 33–78), and 10 healthy donors (HD) (median age, years ± SD: 29 ± 12.04, range 23–59) with an unknown history of any liver disease were included in this exploratory study. Patients were treated according to standard therapeutic protocols, receiving imaging evaluation in frame of routine surveillance. Patients and HD were recruited in 2019 at the University Hospital, LMU Munich. The study was approved by the local ethical board and conducted in accordance with the Declaration of Helsinki [52]. Before entering the study, all participants gave their written informed consent.

### 4.2. Image Acquisition

Patients underwent MRI examination on a 1.5 T MR-scanner (Magnetom Avanto or Magnetom Aera, Siemens, Erlangen, Germany), using an 18-channel phased-array body coil. Patients received an injection of 0.1 mL/kg gadoxetic acid (Primovist, Bayer Schering Pharma AG, Berlin, Germany) unless contraindicated. The standard liver-MRI protocol in our institution consisted of precontrast three dimensional volumetric T1-weighted gradient echo sequences (GRE); unenhanced T1-w in- and opposed-phase; T1-w GRE sequences after injection of intravenous contrast in late arterial (individually timed according to real time bolus tracking), portovenous (70–80 s) and venous (180 s) phases; all axial, slice thickness 5 mm; followed by T2-weighted turbo spin echo sequences with and without fat suppression imaging; diffusion-weighted imaging (DWI, b-values 50, 400, 800) with apparent diffusion coefficient mapping and in the hepatobiliary phase 20 min after the injection of contrast media T1-w GRE 3D sequences in coronal and axial planes.

### 4.3. Image Analysis

Two board-certified radiologists, blinded to the genetic profiling results, evaluated all MRI scans available for each patient for the presence of any lesions. The criteria for HCC diagnosis were >1 cm lesion with wash-in and -out or more than a 50% diameter increase within 6 months. Lesions without enhancement in arterial phase but hypointensity in venous and hepatobiliary were called early HCC; lesions with no wash-in or wash-out, but hypointensity in the hepatobiliary phase were called high-grade dysplastic nodules (HGDN) [33,53].

### 4.4. DNA Isolation, Library Preparation and Next-Generation Sequencing (NGS)

cfDNA was isolated and NGS was performed as previously described [23]. In brief, peripheral blood (5 mL) was drawn into EDTA tubes (Sarstedt AG, Nümbrecht, Germany) and centrifuged 30 min after the blood was drawn (3000 rpm, 5 min, 4 °C) to collect plasma, which was aliquoted and stored at −80 °C until further use. DNA extraction, quality control and NGS were performed at Eurofins Genomics GmbH (Konstanz, Germany). Due to high genomic DNA contamination or due to an amount of cfDNA below the detection level, 7 samples (3 from the HD group and 4 from the LC patients group) failed the quality control test and were excluded from any further analysis. Based on the GATCLiquid Oncopanel All-in-One (Eurofins Genomics), targeted enrichment of 597 selected cancer-relevant genes was run using gene-specific probes [54,55]. To distinguish somatic from germline mutations, genomic DNA extracted from whole blood was analyzed in parallel.

### 4.5. Statistical Analysis

All statistical analyses were performed using IBM SPSS Statistics 21.0.0 (IBM Corporation, New York, NY, USA). Results for numerical data are given as a median together with a minimum and a maximum of the sample (i.e., range). Patients were clustered in two groups corresponding to high and low cfDNA concentration (high cfDNA = over the median, low cfDNA = below the median). The association between cfDNA levels, variants and the patients’ clinical characteristics were evaluated using the Fisher’s exact test. cfDNA levels were compared between patient groups using the non-parametric Mann–Whitney U test. All tests were carried out two-sided. Due to the low sample size, no alpha adjustment was made. All statistical tests are interpreted at a significance level of alpha = 5% with the according results considered exploratory.

## 5. Conclusions

In the era of personalized medicine and integrated diagnostics, our data strengthens the vision that cell-free DNA can support the interpretation of the imaging data and play a key role as a novel companion diagnostic test to monitor early HCC development from liver cirrhosis.

## Figures and Tables

**Table 1:** Clinicopathological characteristics of the patients.

Variables		Total
Patients		40
Gender	male	32
	female	8
Age at inclusion	median ± SD (range)	56 ± 10.41 (33–78)
	<50 years	8
	≥50 years	32
Etiology	AIH	2
	AC	15
	AC/HCV	3
	AC/AIH	1
	Viral	3
	NASH/PBC	1
	PBC/AIH	2
	PSC	5
	B–CS	2
	medication	2
	other	3
BMI (kg/m^2^)	median (range)	27.26 (18.62–41.52)
Child-Pugh score	A (5–6)	12
	B (7–9)	23
	C (10–15)	5
MELD score	<15	20
	≥15	20
AFP (ng/mL)	<10	27
	≥10	1
	n.a.	12
Diabetes	yes	9
	no	27
Portal vein thrombosis	yes	4
	no	36
(Chronic) renal failure	yes	16
	no	23
	n.a.	1
Ascites	yes	21
	no	19
Varices	yes	35
	no	5
Reasons for MRI	Transplantation evaluation	1
	PSC	5
	Portal vein thrombosis	2
	Suboptimal ultrasound	2
	TIPS evaluation	2
	Cystic pancreatic lesion	1
	B–CS	1
	Post-transplant control	1

AC, alcoholic cirrhosis; AFP, alpha fetoprotein; AIH, autoimmune hepatitis; B–CS, Budd–Chiari syndrome; BMI, body mass index; HCV, hepatitis C virus; MELD, model for end-stage liver disease; n.a., not available; NASH, non-alcoholic steato-hepatitis; PBC, primary biliary cholangitis; PSC, primary sclerosing cholangitis; TIPS, transjugular intrahepatic porto-systemic shunt.

**Table 2:** Genetic variants identified in plasma cfDNA. Chromosome location, amino acid (AA) change, codon change and variant type are indicated.

Gene	Chr:Position	Codon Change	AA Change	Variant Type
*ABCB1*	Chr.7:87531302	c.2677T > G	p.S893A	frameshift
		c.2485T > G	p.S829A	
*AC055811.2*	Chr17:17216394	c.119insC	-	frameshift
*ASXL1*	Chr.20:32434638	c.1919insG	p.G641fs	frameshift
		c.1934insG	p.G646fs	
*AXIN2*	Chr.17:65536466	c.1799delG	p.G600fs	frameshift
		c.1994delG	p.G665fs	
*BAX*	Chr.19:48955713	c.121insG	p.E41fs	frameshift
		c.69insG	p.R24fs	
		c.70insG	p.E24fs	
*BRAF*	Chr.7:140777014	c.1592G > T	p.W531L p.W138L	SNV
		c.413G > T		
		c. * 1042G > T		
*BRCA2*	Chr13:32333283	c.1813delA	p.I605fs	frameshift
*BRCA2*	Chr13:32339421	c.5073delA	p.K1691fs	frameshift
*CD3EAP*	Chr.19:45409478	c.1516C > A	p.Q506K	SNV
		c.1510C > A	p.Q504K	
*CHD2*	Chr15:93002203	c.4173insA	p.Q1392fs	frameshift
*CHEK2*	Chr22:28725242	c. 5731G > A	-	SNV
		c. * 424 + 1G > A	-	
		c.220 + 1G > A	-	
		c.474 + 1G > A	-	
		c.5731G > A	-	
		c.6 + 1G > A	-	
		c.444 + 1G > A	-	
*CYP2B6*	Chr19:41006936	c.516G > T	p.Q172H	SNV
*CYP2B6*	Chr19:41009358	c.785A > G	p.K262R	SNV
*ERCC1*	Chr19:45409478	c.197G > T	-	SNV
*FLCN*	Chr17:17216394	c.1285insC	p.H429fs	frameshift
*HNF1A*	Chr12:120994314	c.685insC	p.Q229fs	frameshift
		c.872insC	p.G292fs	
*MSH6*	Chr2:47803500	c.165delC	p.F56fs	frameshift
		c.3261delC	p.F1088fs	
		c. * 2608delC	-	
		c.2871delC	p.F958fs	
		c.2355delC	p.F786fs	
*MPL*	Chr.43338725	c.391 + 5G > C	-	SNV
		c.370 + 5G > C	-	
*NBN*	Chr8:89937066	c. * 2067C > T	-	SNV
*PTEN*	Chr.10:87933154	c.395G > A	p.G132D	SNV
*SLCO1B1*	Chr12.21178615	c.521T > C	p.V174A	SNV
*WRN*	Chr8:31058454	c.15delA	p.K5fs	frameshift

* translation termination (stop) codon.

**Table 3:** Molecular profiles of cfDNA in liver cirrhosis (LC) patients. Single nucleotide variant (SNV) (in red) and insertions and deletions (InDels) (in blue) are shown in descending order with respect to the mutation recurrence rate.

Patients (*n* = 36)																																				Variant	%
																																				HNF1A	28
																																				BAX	*25*
																																				ASXL1	19
																																				CHD2	11
																																				CYP2B6	5
																																				FLCN	5
																																				NBN	5
																																				ABCB1	3
																																				AC055811.2	3
																																				AXIN2	3
																																				BRCA2	3
																																				BRAF	3
																																				CD3EAP	3
																																				CHEK2	3
																																				ERCC1	3
																																				MSH6	3
																																				MPL	3
																																				PTEN	3
																																				SLCO1B1	3
																																				WRN	3

**Table 4:** Distribution of variants among the patients who received an MRI. Twenty-seven patients received an MRI and were therefore eligible for a scan re-evaluation. In 8 patients no variants in cfDNA were detected and the presence of lesions was also excluded by the second evaluation. On the contrary, positive results for variants in different combinations were detected in 6 patients resulting in the new assessment of discovered lesions that were then classified as high-grade dysplastic nodules (HGDN), early hepatocellular carcinoma (HCC) or HCC.

Patient ID	Variants Detected by NGS	Second MRI Evaluation
	none	no lesion
	none	no lesion
	none	no lesion
	none	no lesion
	none	no lesion
	none	no lesion
	none	no lesion
	none	no lesion
	HNF1A	no lesion
	HNF1A	no lesion
	HNF1A	no lesion
	HNF1A; BAX	no lesion
	BAX	no lesion
	CHD2; CYP2B6	no lesion
	BRCA2; MSH6	no lesion
	ASXL1; CHD2	no lesion
	CYP2B6; WRN	no lesion
	ASXL1	no lesion
	CD3EAP; ERCC1; NBN	no lesion
	AC055811.2; FLCN	no lesion
	BAX; ABCB1; MPL; SLCO1B1	no lesion
92381	BAX	early HCC
92387	HNF1A	early HCC
92396	HNF1A	HGDN
92502	BRAF; NBN; PTEN	HCC
92505	BAX; ASXL1; CHD2	HCC
92507	HNF1A; ASXL1	HCC

**Table 5:** Second MRI evaluation. Nine patients were eligible for a second MRI evaluation in frame of the follow-up study. The MRI was run after 17 ± 2 months from the first MRI re-evaluation and next generation sequencing (NGS) analysis. In 7 patients no new lesions were discovered, while in two patients (ID 92381 and 92502) new HCC lesions were found, which had been undetectable at the time of the variant analysis.

Patient’s ID	Variants	Second MRI Re-Evaluation
92379	none	no new lesion
92381	BAX	Early HCC (confirmed) HCC (newly discovered)
92382	AC055811.2; FLCN	no new lesion
92389	none	no new lesion
92396	HNF1A	HGDN (confirmed)
92397	HNF1A	no new lesion
92500	CYP2B6; WRN	no new lesion
92502	BRAF; NBN; PTEN	HCC (confirmed) HCC (newly discovered)
92504	ASXL1	no new lesion

**Table 6:** Comparison of cfDNA amounts between sub-cohorts with liver cirrhosis (LC), malignant lesions and healthy donors (HD). A significant difference between cfDNA level in LC patients and HD (*p* < 0.005) and between cfDNA level in patients with malignant lesions (including HGDN and early HCC) and HD (*p* < 0.005) was found. No significant difference (*p* = 0.467) was found when the level of cfDNA from the patients with HGDN, early HCC or HCC was compared to the level of cfDNA in cirrhotic patients.

Sub-Cohort	cfDNA (ng/µL)	Sub-Cohort	cfDNA (ng/µL)	
	Mean ± SD		Mean ± SD	*p* Values
LC (*n* = 36)	42.27 ± 42.42	HD (*n* = 7)	8.46 ± 1.47	<0.005
LC (*n* = 30)	39.58 ± 42.77	HD (*n* = 7)	8.46 ± 1.47	<0.005
Malignant lesion (*n* = 6)	55.69 ± 37.83	HD (*n* = 7)	8.46 ± 1.47	0.007
Malignant lesion (*n* = 6)	55.69 ± 37.83	LC (*n* = 30)	39.58 ± 42.77	0.445

**Table 7:** Association between cfDNA and patients’ characteristics. No significant association was found between the amount of cfDNA and patients’ clinical characteristics, with the only exception of AFP (*p* = 0.024).

Variable	Total (*n* = 36)	Low cfDNA (*n* = 21)	High cfDNA (*n* = 15)	*p* Value
Gender				0.667
female	6	3	3	
male	30	18	12	
Age				0.077
<60 years	25	12	13	
>60 years	11	9	12	
Etiology				0.500
AC positive	18	12	6	
AC negative	18	9	9	
BMI (kg/m^2^)				0.499
<25	14	7	7	
>25	22	14	8	
Child-Pugh score				0.320
≤7	16	11	5	
>7	20	10	10	
MELD				0.500
<15	20	13	7	
≥15	16	8	8	
AFP (ng/mL)				0.024
≤7	25	12	13	
>7	1	0	1	
diabetes				0.705
no	27	15	12	
yes	9	6	3	
Portal vein thrombosis				1.000
no	32	19	13	
yes	4	2	2	
(Chronic) renal failure				0.728
no	20	11	9	
yes	15	10	5	
Ascites				0.516
no	17	11	6	
yes	19	10	9	
Varices				1.000
no	4	2	2	
yes	32	19	13	

**Table 8:** Distribution of variants with respect to etiologies.

Variant	AC	HBV/HCV	NASH	PSC	B–CS	PBC/AIH	CPA	CPB	CPC
*HNF1A*									
*BAX*									
*ASXL1*									
*CHD2*									
*CYP2B6*									
*FLCN*									
*NBN*									
*AC055811.2*									
*AXIN2*									
*ABCB1*									
*BRCA2*									
*BRAF*									
*CD3EAP*									
*CHEK2*									
*ERCC1*									
*MSH6*									
*MPL*									
*PTEN*									
*SLCO1B1*									
*WRN*									

AC, alcoholic cirrhosis; AIH, autoimmune hepatitis; B–CS, Budd–Chiari syndrome; CP (A/B/C), Child Pugh; HBV, hepatitis B virus; HCV, hepatitis C virus; non-alcoholic steato-hepatitis; PBC, primary biliary cholangitis; PSC, primary sclerosing cholangitis; detection of a variant in the presence of a specific etiology is indicated in grey.

## Data Availability

The data presented in this study are available on request from the corresponding author. The data are not publicly available due to ethical restrictions.

## Supplementary Materials

The following are available online at https://www.mdpi.com/2072-6694/13/3/521/s1, Supplementary File 1, Figure S2. Patient 92381: Venous phase (left upper) and hepatobiliary phase (left lower) MRI showed a hypointense lesion in segment 5, Figure S3. Patient 92387: Appearance of an early HCC lesion with hypointensity on venous (not shown) and hepatobiliary (left image) phases without hyper-vascularity after the detection of mutated variants, which doubled in size in 6 months (right image), Figure S4. Patient 92396: Hepatobiliary phase MRI showed a hypointense lesion in segment 7. The lesion was not visible in dynamic series (not shown), Figure S5. Patient 92502: Consecutive arterial (lower row) and venous (upper row) phase show progression of a small hyper vascular lesion into HCC and development of washout appearance after detection of mutated variants, Figure S6. Patient 92505: Follow-up hepatobiliary phase images show progression in size of a dysplastic nodule (lesion was hyper intense also in native T1 imaging, not shown) and development of typical HCC enhancement pattern with arterial wash-in and venous wash-out (upper panel, CT images), Figure S7. Patient 92507: Follow-up hepatobiliary phase images show progression in size of a dysplastic nodule (lesion was hyper intense also in native T1 imaging, not shown) and development of typical HCC enhancement pattern with arterial wash-in and venous wash-out (upper panel, subtraction images), Figure S8. Patient 92502: Consecutive arterial (lower row) and venous (upper row) phase show progression of a small hyper vascular lesion into HCC and development of washout appearance after detection of mutated variants.
