# Supplementary material for: Circulating Cell-Free DNA Combined to Magnetic Resonance Imaging for Early Detection of HCC in Patients with Liver Cirrhosis
Source: Cancers (Basel). 2021 Jan 29;13(3):521. doi: 10.3390/cancers13030521 (PMC7866376; doi:10.3390/cancers13030521)
Supplement: Supplementary file 1 [file cancers-13-00521-s001.zip › cancers-1054592-supplementary/Supplementary Figure 2-8.docx]

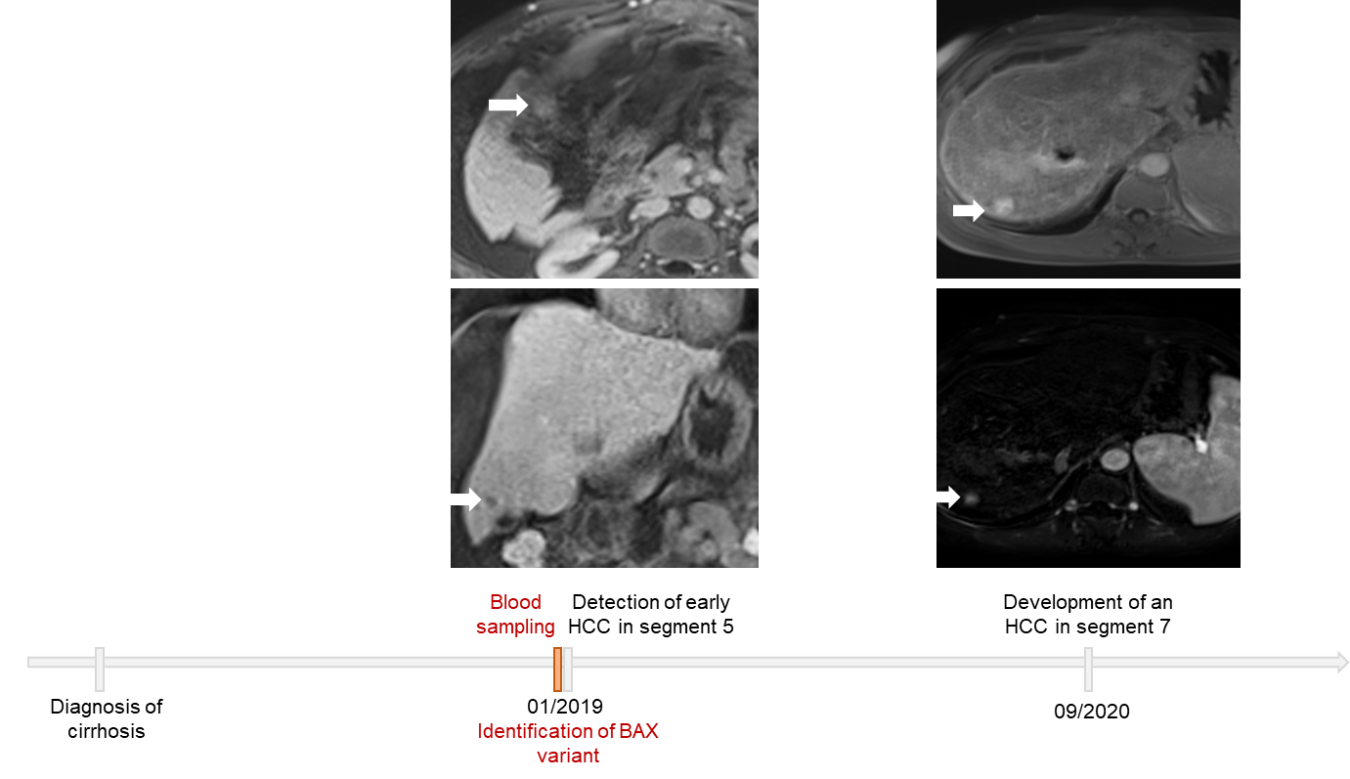


**Figure S2.** Patient 92381: Venous phase (left upper) and hepatobiliary phase (left lower) MRI showed a hypointense lesion in segment 5. The lesion did not show hypervascularity in arterial phase (not shown). Lesion was classified as early HCC. NGS evidenced a variant in the BAX gene. Follow-up MRI showed minimal size increase in early HCC (not shown), and also the development of an HCC lesion in segment 7, which was hyperintense in all sequences (hepatobiliary phase, right upper) yet with arterial hypervascularity confirmed in subtracted images (right lower).


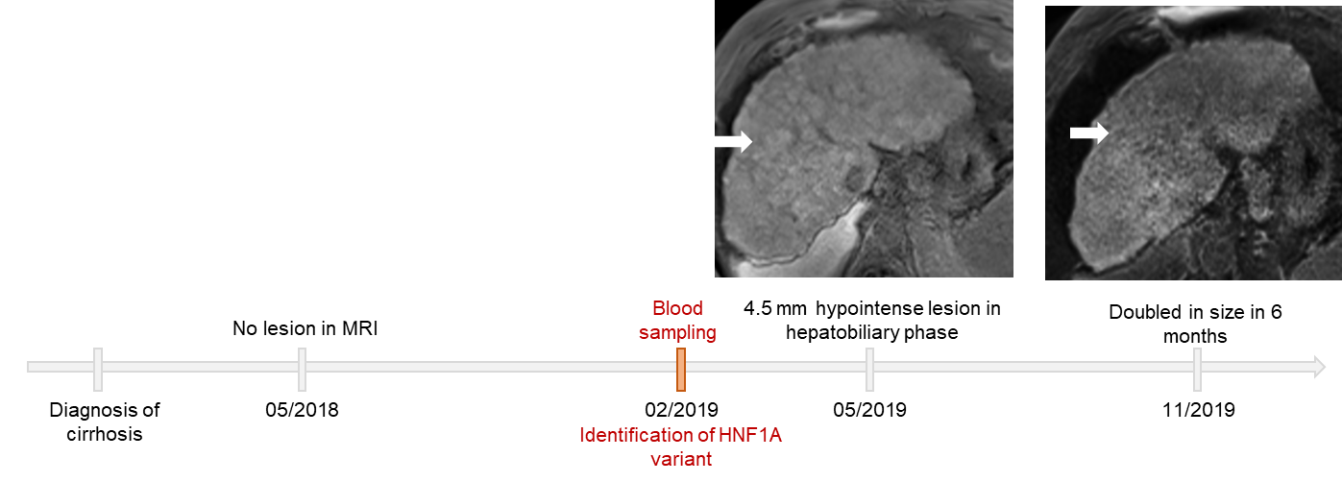


**Figure S3.** Patient 92387: Appearance of an early HCC lesion with hypointensity on venous (not shown) and hepatobiliary (left image) phases without hyper-vascularity after the detection of mutated variants, which doubled in size in 6 months (right image). NGS evidenced a variant in the HNF1A gene.


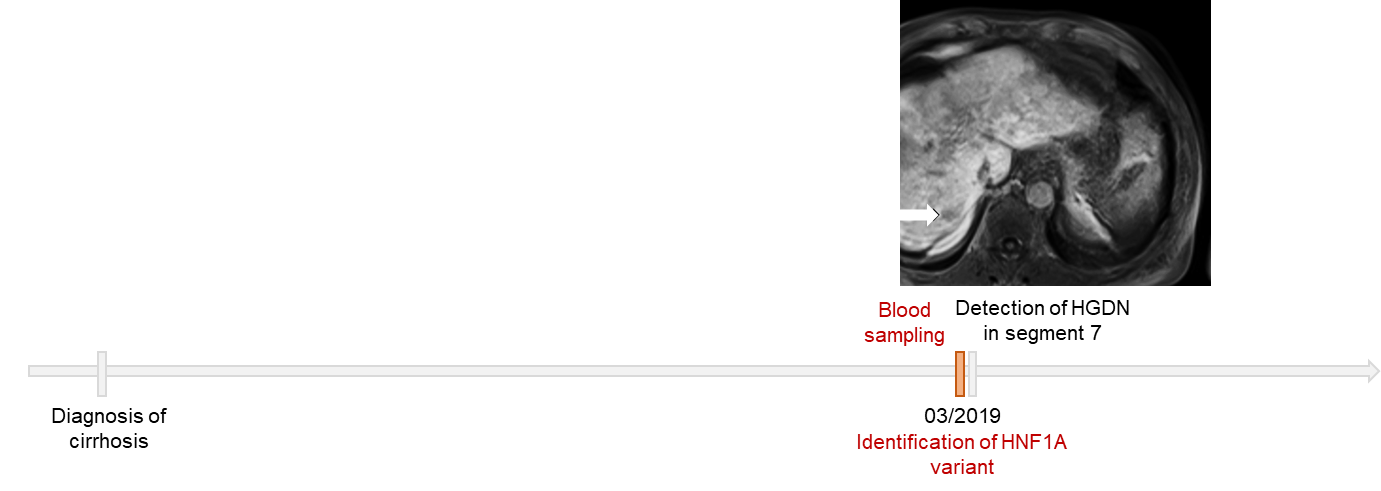


**Figure S4.** Patient 92396: Hepatobiliary phase MRI showed a hypointense lesion in segment 7. The lesion was not visible in dynamic series (not shown). Lesion was classified as HGDN. NGS evidenced a variant in the HNF1A gene.


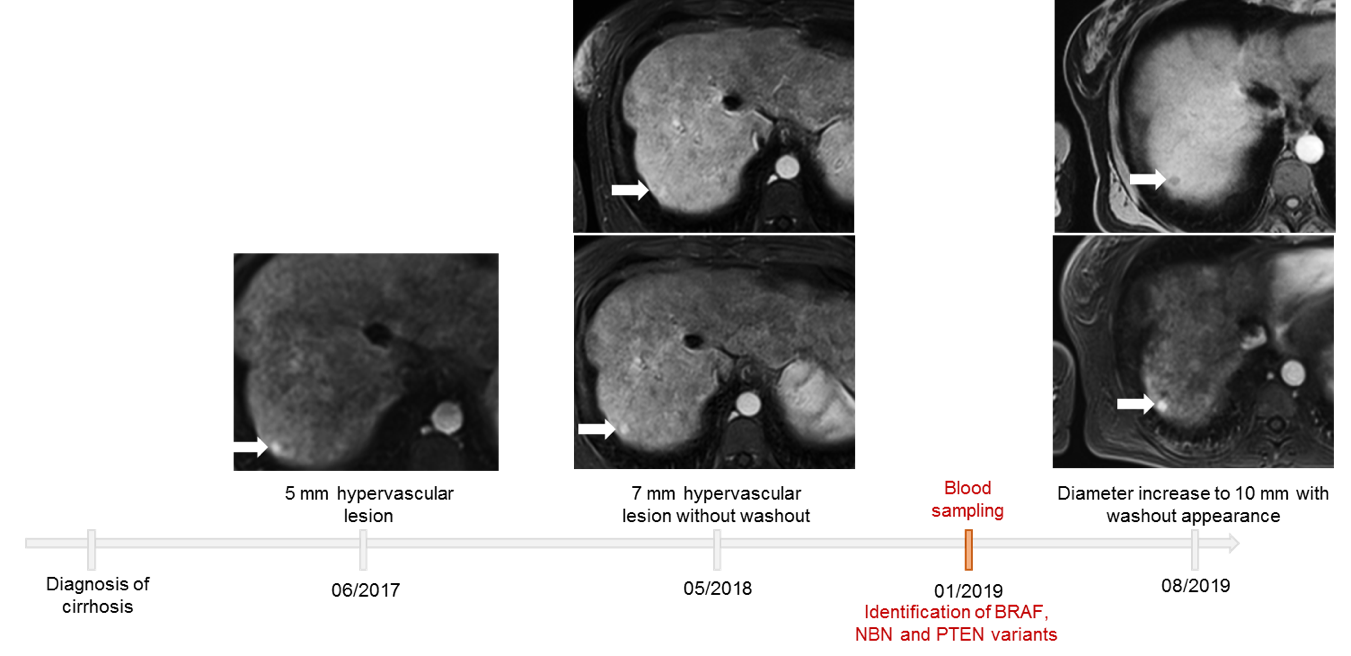


**Figure S5.** Patient 92502: Consecutive arterial (lower row) and venous (upper row) phase show progression of a small hyper vascular lesion into HCC and development of washout appearance after detection of mutated variants. NGS analysis evidenced variants in the BRAF, NBN and PTEN genes. Follow-up MRI image during preparation to liver transplantation revealed five new HCC lesions developed arterial hypervascularity (far right images).

**Figure S6.** Patient 92505: Follow-up hepatobiliary phase images show progression in size of a dysplastic nodule (lesion was hyper intense also in native T1 imaging, not shown) and development of typical HCC enhancement pattern with arterial wash-in and venous wash-out (upper panel, CT images). Subtraction imaging in previous MRIs did not reveal hyper vascularity (not shown). NGS analysis evidenced variants in the BAX, ASXL1 and CHD2 genes.


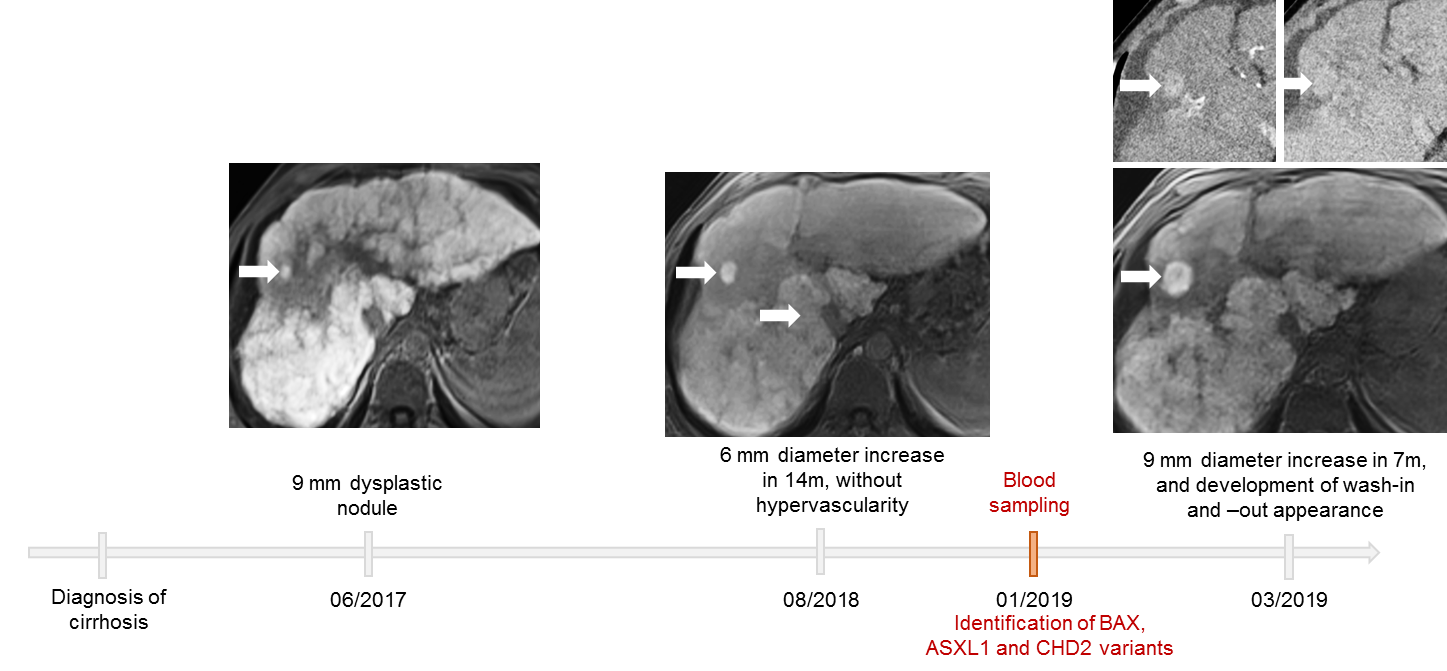


**Figure S6.** Patient 92505: Follow-up hepatobiliary phase images show progression in size of a dysplastic nodule (lesion was hyper intense also in native T1 imaging, not shown) and development of typical HCC enhancement pattern with arterial wash-in and venous wash-out (upper panel, CT images). Subtraction imaging in previous MRIs did not reveal hyper vascularity (not shown). NGS analysis evidenced variants in the BAX, ASXL1 and CHD2 genes.


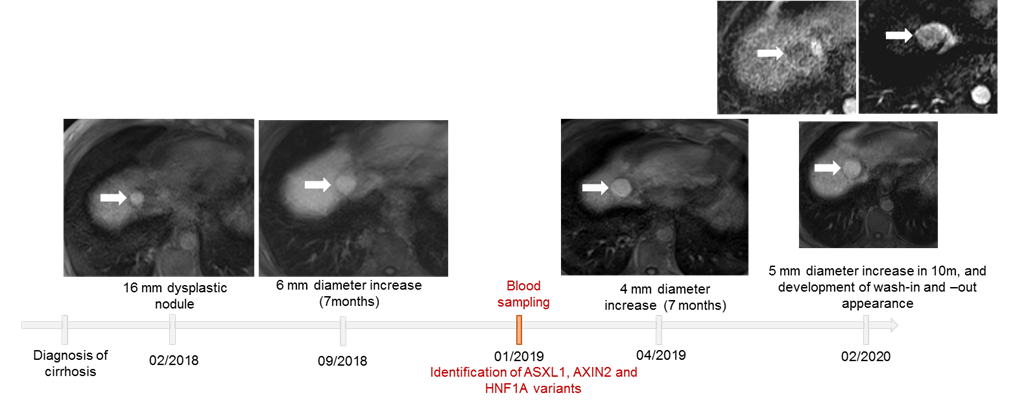


**Figure S7.** Patient 92507: Follow-up hepatobiliary phase images show progression in size of a dysplastic nodule (lesion was hyper intense also in native T1 imaging, not shown) and development of typical HCC enhancement pattern with arterial wash-in and venous wash-out (upper panel, subtraction images). NGS analysis evidenced variants in the ASXL1, AXIN2 and HNF1A genes.


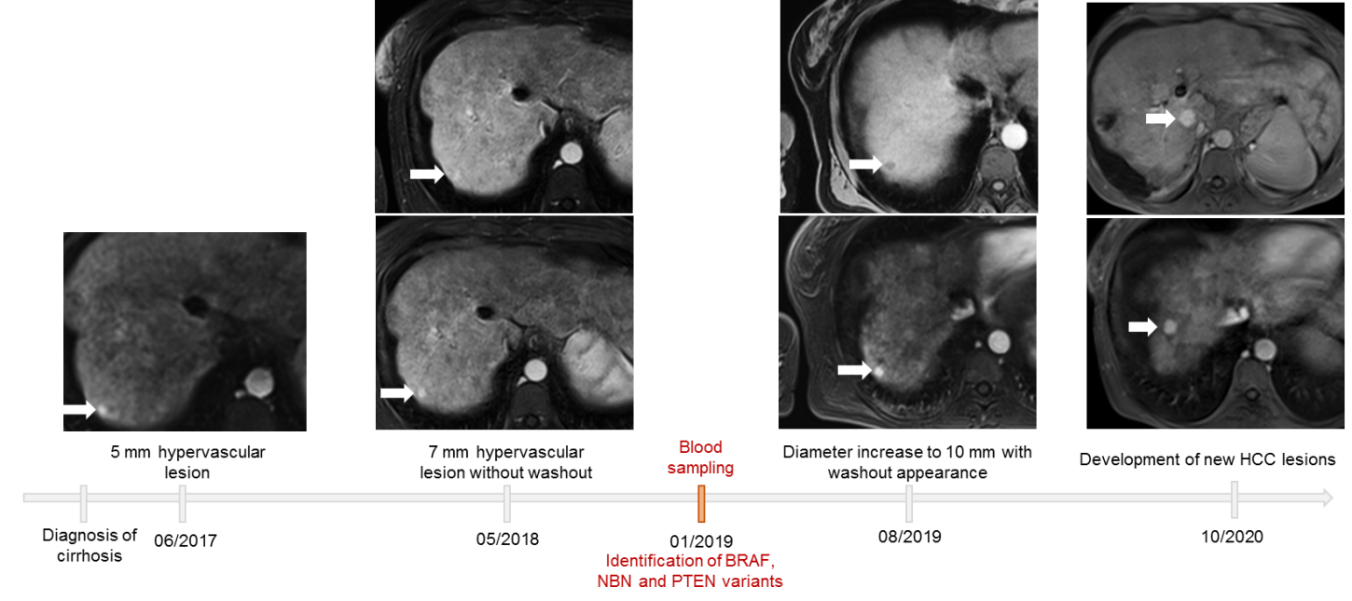


**Figure S8.** Patient 92502: Consecutive arterial (lower row) and venous (upper row) phase show progression of a small hyper vascular lesion into HCC and development of washout appearance after detection of mutated variants. Follow-up MRI image during preparation to liver transplantation revealed five new HCC lesions (far right images).
