# Supplementary material for: Circulating Cell-Free DNA Combined to Magnetic Resonance Imaging for Early Detection of HCC in Patients with Liver Cirrhosis
Source: Cancers (Basel). 2021 Jan 29;13(3):521. doi: 10.3390/cancers13030521 (PMC7866376; doi:10.3390/cancers13030521)
Supplement: Supplementary file 1 [file cancers-13-00521-s001.zip › cancers-1054592-supplementary/Supplementary File 1.docx]

**Supplementary File 1**

Case reports

Patient 92381 (female, 56 years old, affected by Budd-Chiari syndrome, diagnosed with ovarian cancer one month after study inclusion) was included in the study on January 2019. On the same day the blood was collected to perform NGS and the patient received a MRI, which evidenced a hypointense lesion (<10 mm) in venous and hepatobiliary phases without arterial hypervascularity in segment 5, while cfDNA analysis revealed the presence of an InDel mutation in the BAX gene. The lesion was classified as early HCC (Supplementary Figure 2).

Patient 92387 (male, 50 years old, cirrhosis associated with alpha1-antitrypsin deficiency) was included in February 2019. NGS revealed an InDel mutation in the HNF1A gene. Nine months before, the patient received a MRI as part of cirrhosis monitoring, which did not detect any malignant lesion. Three months after blood analysis (May 2019), the patient was checked again by MRI and still no lesion was visible. However, during a second follow up (November 2019) a suspicious lesion of 8 mm was identified; because of the size, the lesion was nevertheless still not considered as an HCC. Running a re-evaluation of the scan obtained in May 2019, which was initially evaluated as negative, the radiologists were able this time to detect the same nodule as a small (4.5 mm) hypointense lesion in the venous phase. Presence of wash-out led to a diagnosis of early HCC, i.e. a lesion without enhancement in arterial phase but hypointensity in venous and hepatobiliary. Noteworthy, the variant carried by HNF1A was detectable already three months earlier than the second negative MRI run in May 2019. The patient was listed for orthotopic liver transplantation since May 2018 and underwent organ transplantation in December 2019. A second liver transplantation was urgently necessary in December 2019 due to allograft dysfunction. Unfortunately, the patient deceased in January 2020 with acute liver failure due to allograft dysfunction also after the second organ transplantation (Supplementary Figure 3).

Patient 92396 (male, 55 years old, affected by AC) was included in March 2019. cfDNA analysis revealed the presence of an InDel mutation in the HNF1A gene. The MRI scans revealed a high-grade dysplastic nodule (HGDN) in segment 7 with hypointensity in the hepatobiliary phase without identification on dynamic series (Supplementary Figure 4).

Patient 92502 (female, 53 years old, affected by cirrhosis due to autoimmune hepatitis and primary biliary cholangitis) was included in January 2019. NGS analysis of plasmatic cfDNA identified variants in the BRAF, NBN and PTEN genes. MRI performed 7 months later (August 2019) evidenced a lesion of 10 mm with hypervascularity and washout appearance, therefore classified as HCC. Interestingly, the MRI performed in 2017 and in 2018 evidenced the same lesion as 5 mm and 7 mm, respectively, which back then were not classified as HCC due to the small size (Supplementary Figure 5).

Patient 92505 (male, 50 years old, affected by AC and chronic HCV infection) was included in January 2019 and cfDNA analysis revealed InDel variants in the BAX, ASXL1 and CHD2 genes. Three months later (March 2019) MRI revealed progression in size and development of arterial wash-in and venous wash-out of a dysplastic nodule, which led to the diagnosis of HCC. The same lesion was already identified in June 2017 with confirmed minimal size increase in August 2018. At that time the lesion was not classified as HCC and no biopsy was conducted, because despite the size ≥ 10 mm, there was absence of wash-in and wash-out appearance. After diagnosis with HCC, the patient was treated with radiofrequency ablation and at follow up he did not show any sign of recurrence of malignancy (Supplementary Figure 6).

Patient 92507 (male, 66 years old, affected by AC) was included in January 2019 and cfDNA analysis revealed variants in the AXIN2, ASXL1 and HNF1A genes. MRI, performed before (February 2018 and September 2018) and after (April 2019) blood collection, evidenced a hyperintense lesion on native T1 series which was classified as a dysplastic nodule and therefore followed with regular intervals. However, as evidenced during the last MRI performed in February 2020, the lesion progressed to 31 mm and developed arterial hypervascularity and wash-out appearance, consequently the lesion was classified as HCC. Due to the location of the disease, CT - guided brachytherapy was chosen as the loco-regional therapy strategy, which has already performed at the time of writing with a follow-up imaging showed no sign of recurrence (Supplementary Figure 7).
